# A comparative study of effects of curcumin and its nanoparticles on the growth, immunity and heat stress resistance of Nile tilapia (*Oreochromis niloticus*)

**DOI:** 10.1038/s41598-023-29343-z

**Published:** 2023-02-13

**Authors:** Heba M. Abdel-Ghany, Doaa M. El-Sisy, Mohamed El-S. Salem

**Affiliations:** National Institute of Oceanography and Fisheries, NIOF, Cairo, Egypt

**Keywords:** Physiology, Zoology, Ocean sciences

## Abstract

This study evaluated the effects of dietary supplementation with free- or nano-curcumin on the growth performance, immune status and heat stress resistance of Nile tilapia (*Oreochromis niloticus*). Seven isonitrogenous (28% protein) and isocaloric (445 kcal/100 g DM) diets were prepared. Six diets were supplemented with three levels of nano-curcumin (50 (CN50), 100 (CN100), 200 (CN200) mg kg^−1^ diet) or free-curcumin (50 (C50), 100 (C100), 200 (C200) mg kg^−1^ diet), and the control diet was left without an additive (CON). Fish (13.54 ± 0.32 g) (mean ± SD) fed the experimental diets for 65 days. Following the feeding trial, the fish were exposed to the acute heat stress by gradually raising the water temperature from 25 to 40 °C within 3 h. The fish were then exposed to 40 °C for 4 h. Results revealed the superiority of nano-curcumin over its free-form in enhancing the growth performance, with the highest results obtained at CN100, followed by CN200. Only heat stress, not the experimental diets, increased the platelets, mean corpuscular volume (MCV), mean corpuscular hemoglobin (MCH), leukocytes and neutrophils count, while lymphocytes decreased. The CN50 and CN100 groups showed lower activity of liver enzymes (alanine aminotransferase (ALT) and aspartate aminotransferase (AST)) than the other treatments, while C200 gave the highest activity of these enzymes. The highest immunoglobulin (IgM) levels were detected in CN100, CN200, C100 and C200, followed by CN50. The C200 group showed higher levels of complement 3 and complement 4 (C3 and C4, respectively) than the other treatments. The C50 and CON groups gave the lowest values of IgM, C3 and C4. Cortisol levels were significantly lower in the CN50 and CN100 groups compared to the other groups. After the heat stress, ALT, AST, IgM, C3, C4, cortisol and glucose increased. Thus, nano-curcumin is more effective than its free-form in enhancing the resistance to heat stress, inducing innate immunity, lowering the stress indicators and promoting growth performance of Nile tilapia with the best concentration at 100 mg kg^−1^ diet.

## Introduction

The second-most common fish species produced globally after carp is the Nile tilapia (*Oreochromis niloticus*)^1,2^. Tilapia are currently cultured in more than 120 countries worldwide^2^. Global tilapia production bounced from less than 0.5 million metric tons (MMT) in the early 1990s to 6.03 MMT in 2018, with an average annual growth rate of 13.5%^2^. However, the intensive culture of tilapia makes it sensitive to various stress factors, such as heat stress (HS), a major consequence of the global warming^3^. Although Nile tilapia can tolerate a broad range of temperatures (the optimum temperature range is 25–28 °C), water temperatures above the optimum range may suppress the immune system and damage its antioxidant response^4,5^. HS suppresses the immune system through cell mediated and humoral immune responses^6^. For instance, HS has been found to reduce the level of immunoglobulins (IgG and IgM)^7^. Primary indicators of the immune response (white blood cells, red blood cells, hemoglobin and glucose) are also affected during HS^8^. Blunt snout bream (*Megalobrama amblycephala* Yih) showed increased lysozyme, alternative complement activities, total protein and IgM levels after being exposed to heat stress for 6 h, thereafter, these parameters decreased^9^. Consequently, heat stress can adversely affect fish health, leading to growth regression and high fish mortality^10^. Strengthening the defense mechanisms of fish through the administration of immuno-stimulants in the diet has become a priority for controlling stress factors in aquaculture^11^. Among various immuno-stimulants, medicinal herbs contain chemical constituents that enhance the immunity via specific or non-specific pathways, making the animal more resistant to external stressors^12^.

Curcumin is an orange-yellow, hydrophobic, bioactive herbal ingredient and polyphenolic compound extracted from turmeric (*Curcuma longa*). The major active constituents of curcumin are diferuloylmethane (77%), demethoxycurcumin (17%), and bisdemethoxycurcumin (6%)^13^. Surprisingly, curcumin possesses antiviral, antifungal, anti-inflammatory, antioxidant and anticancer properties^14–17^. Thus, curcumin can stimulate the innate immune system in fish^18,19^. Several studies have shown the beneficial effects of curcumin as a potential immunostimulant and antioxidant candidate in diets of various fish species^20–26^. However, poor water solubility and bioavailability limit the practical applications of curcumin^13^.

Recently, nano-curcumin has been developed to avoid the poor delivery properties of curcumin and enhance its therapeutic effects^27^. Unlike curcumin, which forms clumps because of its low solubility in water, nano-curcumin completely dissolves in an aqueous medium and does not form any clumps due to the presence of zeta potential^27^. In comparison to free-curcumin, nano-curcumin demonstrated higher permeability, longer circulation, and better systemic bioavailability in plasma and tissues^28–30^.

According to Jiang et al.^31^ curcumin loaded in nanospheres stimulated its antioxidant and immunological activities. These effects may be linked to the reduction of particles to a nano-metric scale, which ensures a sustainable release of the active materials and increases the bioavailability and levels of molecular dispersion^31^. Although no studies have been done to compare the effect of nano-curcumin to that of its free form on fish, it has recently been tested on birds such as Japanese quail (*Coturnix japonica*)^32^. Marchiori et al.^32^ found that nano-encapsulated curcumin improved the performance of Japanese quails (*Coturnix japonica*) at a threefold lower dose than free-curcumin, indicating the potential of nano-materials to improve animal production.

Until now, nano-curcumin has not been tested in fish diets. Therefore, the aim of this study was to evaluate the effects of dietary supplementation with free- or nano-form of curcumin on improving the growth performance, immunity status and heat stress resistance in Nile tilapia.

## Materials and methods

### Experimental diets

Composition and the chemical analysis of the experimental diets are shown in Table 1. Seven isonitrogenous (28% protein) and isocaloric (445 kcal/100 g DM) diets were prepared. Six diets were supplemented with three levels of nano-curcumin (50 (CN50), 100 (CN100), 200 (CN200) mg kg^−1^ diet) or free-curcumin (50 (C50), 100 (C100), 200 (C200) mg kg^−1^ diet), and the control diet was left without an additive (CON). Dry and finely ground ingredients were thoroughly mixed by hand while adding the oils. After that, all the ingredients were kneaded by gradually adding warm water. The dough was pelletized using a meat grinder of a suitable diameter. Diets were dried in the sun and stored at -20 °C in plastic jars.

**Table 1:** Formulation and proximate composition of the experimental diets (g kg^−1^, dry weight) fed to Nile tilapia (*Oreochromis niloticus*).

Ingredients (g kg^−1^)	Diet 1 (CON)	Diet 2 (CN50)	Diet 3 (CN100)	Diet 4 (CN200)	Diet 5 (C50)	Diet 6 (C100)	Diet (C200)
Fish meal (700 g kg^−1^ CP)	50	50	50	50	50	50	50
Soybean meal (440 g kg^−1^ CP)	500	500	500	500	500	500	500
Corn flour	380	380	380	380	380	380	380
Minerals and vitamins^1^	20	20	20	20	20	20	20
Nano-curcumin(mg)	0.0	50	100	200	0.0	0.0	0.0
Curcumin(mg)	0.0	0.0	0.0	0.0	50	100	200
Oils (50% fish oil:50% corn oil)	40	40	40	40	40	40	40
Di-calcium phosphate	10	10	10	10	10	10	10
Proximate composition %							
Protein	28.40	28.23	28.23	28.07	28.05	28.23	28.25
Lipid	6.58	6.62	6.53	6.97	6.67	6.64	6.68
Ash	6.47	6.46	6.53	6.28	6.67	6.48	6.47
Fiber	4.50	4.41	4.38	4.59	4.57	4.46	4.58
Gross energy (Kcal/100 g DM)	444.45	445.35	444.14	446.43	443.28	444.59	444.41

^1^Vitamin premix contains (mg kg^-1^ or IU kg^-1^ of dry vitamin) Vit. A 2.200.000 IU, Vit. D3 1.100.000 IU, Vit. E 1.500 IU, Vit. K 800 mg, Vit. B1 1.100 mg, Vit. B2 200 mg, Vit. B6 2.000 mg, Vit. H 15 mg, Vit. B12 4 mg, Vit. C 3000 mg. Mineral premix contains (mg kg^-1^ of dry mineral powder) iron 160 mg, magnesium 334 mg, copper 21.6 mg, zinc 21.6 mg, selenium 25 mg, cobalt 2.38 mg.

### Fish and the experimental facilities

Nile tilapia (*Oreochromis niloticus*) fingerlings were obtained from a commercial farm near Alexandria and kept in a fiberglass tank (1 m^3^) in the fish rearing laboratory at the National Institute of Oceanography and Fisheries, Alexandria branch, Egypt. One hundred and sixty-eight fish (13.54 ± 0.32 g) were restocked in 21 glass aquaria (70 L aquarium^-1^) at a density of 8 fish per aquarium. Each treatment consisted of 3 replicates (A, B and C). Fish were acclimatized to the aquaria for 2 weeks and were fed on the control diet to adapt to the experimental diets. Fish were fed the experimental diets three times a day until satiety for 65 days. Dechlorinated tap water was provided to each aquarium with a continuous aeration. The amount of feed consumed in each treatment was calculated weekly by the difference in the weight of the food containers before and after feeding. Photoperiod was set at 12 h:12 h light:dark, respectively. The water quality parameters were monitored every week for the duration of the experiment using the Hanna Aquaculture Multi-parameter Photometer, HI83303. Water temperature, dissolved oxygen, ammonia-N and pH were recorded as follows; 25 ± 2.1 °C, 6.4 mg L^−1^, 0.05 mg L^−1^ and 7.7, respectively. After exposure to the heat stress (40 °C), the dissolved oxygen and ammonia-N in the fish tank were 2.1 mg L^−1^, and 0.62 mg L^−1^, respectively. Two hours after feeding, feces were removed by siphoning.

### Ethical declaration

The experiments were performed in accordance with guidelines outlined and approved by the National Institute of Oceanography and Fisheries Committee for Institutional Care of Aquatic Organisms and Experimental Animals (NIOF-IACUC), Egypt.

### Curcumin and nano-curcumin preparation

**Figure 1:**
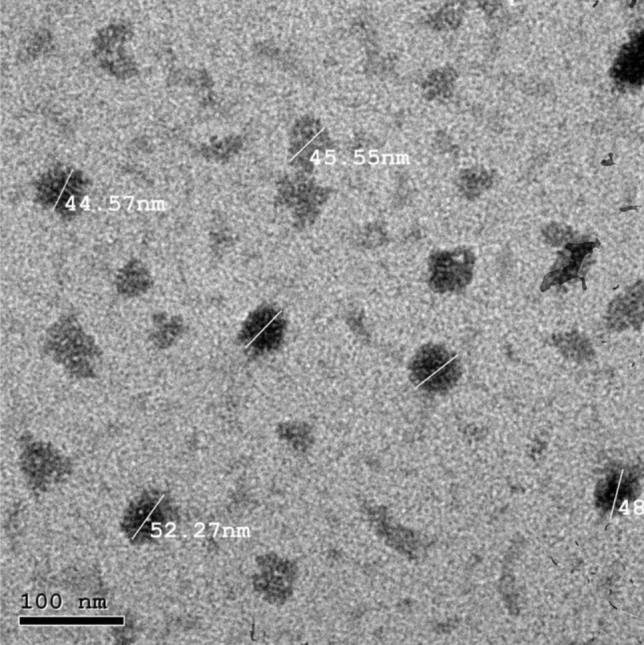
Transmission electron microscope micrograph for as-prepared PVA capped nano-curcumin particles.

**Figure 2:**
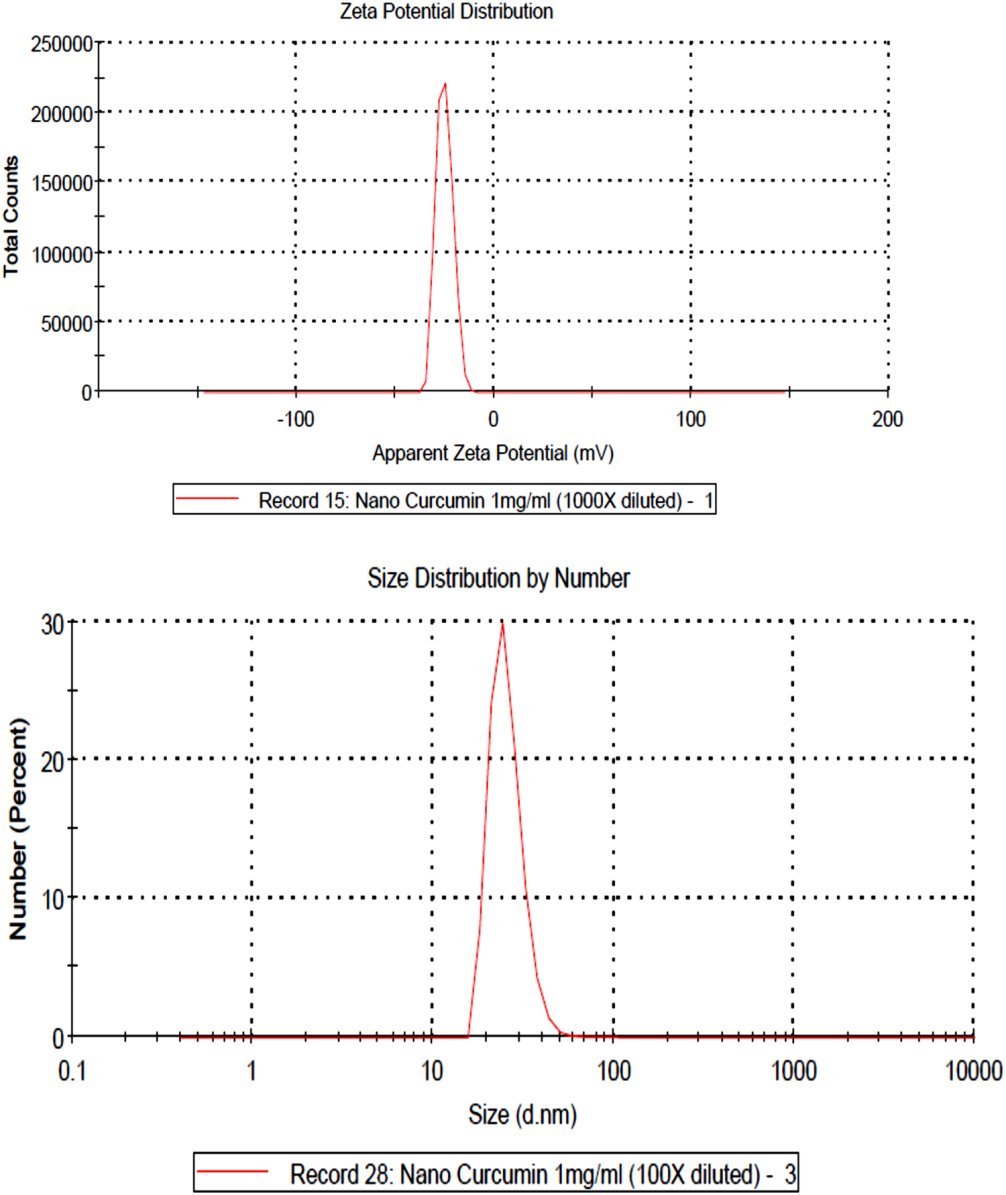
Apparent zeta potential and Zetasizer analysis of nano-curcumin particles.

Curcumin and nano-curcumin were prepared by the research department at NanoTech Egypt for Photo-Electronics and Nanotechnology. Nano-curcumin was prepared through the reduction method as described by Dhivya et al.^33^. UV–Vis absorption spectra were obtained on an Ocean Optics *USB2000* + *VIS–NIR* Fiber optics spectrophotometer. To study the surface morphology and size of the nanoparticles, a high-resolution transmission electron microscope (TEM), JEOL JEM-2100, was used. TEM imaging revealed the nearly spherical shape, smooth surface and uniform size distribution of nearly 50 ± 5.5 nm of nano-curcumin (Fig. 1). Particle size distribution and zeta potential of nano-curcumin particles were determined by the Zetasizer Malvern instrument (Malvern, UK), version 7.13, serial number: MAL 1203718. Zeta potential (mV) and Zeta deviation (mV) were -25.0 and 4.16, respectively (Fig. 2).

### Productive performance

All fish in each tank were collected and weighed before starting and at the terminal of the feeding trial. Growth, feed utilization and other biometric indices were calculated according to the following equations:

$$survival \, rate = \frac{initial \, number \, of \, fish}{{final \, number \, of \, fish}},$$

$$feed \, intake = amount \, of \, consumed \, feed,$$

$$weight \, gain \, \left( {WG} \right)\left( g \right) = final \, weight - initial \, weight,$$

$$specific \, growth \, rate \left( {SGR} \right) \left( {\%\; day^{-1}} \right) = \frac{{ln \left( {final \, weight} \right) - ln \left( {initial \, weight} \right) }}{trial \, duration} \times 100,$$

$$average \, daily \, gain \left( {ADG} \right) \left( {g\,day^{-1}} \right) = \frac{final \, weight\left( g \right) - initial\,\,weight \left( g \right)}{{trial \, duration}},$$

$$feed \, conversion \, ratio \left( {FCR} \right) = \frac{total \, dry \, feed \, intake \left( g \right) }{{ total \, fish \, weight \, gain \left( g \right)}},{\text{ and}}$$

$$protein \, efficiency \, ratio = \frac{WG \left( g \right) }{{ total \, dry \, protein \, intake \left( g \right)}}$$

### Body chemical composition

Before the start of the feeding trial, 10 fish were randomly sampled and frozen at -20 °C for the whole-body biochemistry analysis. At the end of the feeding trial, six fish per treatment were sampled for the whole-body biochemical analysis. All samples were immediately frozen at − 20 °C until analysis. AOAC^34^ methods were followed to evaluate the crude protein, lipid, moisture and ash contents of all samples.

### Blood sampling

Following the feeding experiment, six fish per treatment were sampled for the blood analysis. Clove oil (0.1 ml L^−1^) has been used to anesthetize the fish. Blood samples (1 ml) were collected from the fish caudal vein in plastic microtubes containing an anticoagulant (dipotassium ethylenediaminetetraacetic acid, EDTA) for the preservation of the samples for using in the hematological assay. One mL of blood from each sample was collected into microtubes without an anticoagulant and centrifuged for 10 min at 2500×*g* to separate the serum, which was assigned for the biochemistry assay.

### Hematological assay

Erythrocytes, hematocrit (HCT), hemoglobin (HGB), mean corpuscular volume (MCV), mean corpuscular hemoglobin (MCH), mean corpuscular hemoglobin concentration (MCHC), platelets (PLT), leukocytes and differential counts (neutrophils (NEUT), lymphocytes (LYMPH)), monocytes (MON), eosinophil (EO), basophils (BAS) and immature granulocyte (IG) were counted using automated hemo-cytometer XE 2100 (Advia 2120 & Sysmex, Siemens). All mentioned indices were assessed before and after the heat stress.

### Serum biochemistry

Serum alanine aminotransferase (ALT), serum aspartate aminotransferase (AST), immunoglobulin M (IgM), complements 3 and 4 (C3 and C4, respectively), cortisol and glucose concentrations were analyzed in the serum of blood samples. Glucose was analyzed according to the hexokinase method^35^ using Cobas pack (reference number, 04404483 190) through the Cobas-c 311 analyzer, system-ID 0768316. Cortisol was assayed based on the formation of the respective immune complex method^36^ through the electrochemiluminescence immunoassay “ECLIA” pack (reference number, 11875116 122) using the Cobas-e immunoassay analyzer. ALT analysis was performed using Cobas c pack (reference number, 207649q57 322), Cobas-C 311 analyzer, system ID 0,764,957. ALT was detected according to Bergmeyer et al.^37^ and ECCLS^38^. ALT catalyzed the reaction between L-alanine and 2-oxoglutarate. The formed pyruvate is reduced by NADH in a reaction catalyzed by lactate dehydrogenase (LDH) to form L-lactate and NAD. The rate of the NADH oxidation is directly proportional to the catalytic ALT activity. It is determined by measuring the decrease in absorbance. AST in the sample catalyzed the transfer of an amino group between L-aspartate and 2-oxoglutarate to form oxaloacetate and L-glutamate. The oxaloacetate then reacted with NADH, in the presence of malate dehydrogenase (MDH), to form NAD. This assay followed the recommendations of the IFCC but was optimized according to Bergmeyer et al.^37^ and ECCLS^38^ using Cobas c pack (reference number, 20764949322) through the Cobas-C 311 analyzer. System lD 076494I. IgM was determined based on the principle of immunological agglutination^39^ through the Cobas c 311, system ID 0767883 using Cobas c pack (reference number, 03507190). IgM antibodies and antigens reacted with each other in the sample and formed an antigen/antibody complex. After agglutination, this was measured turbidimetrically at a sub/main wavelength: 700/340. C3 and C4 were determined by forming a precipitate through the addition of a specific antiserum and then they were determined turbidimetrically at a sub/main wavelength: 700/340^39^. C3 was measured through the Cobas c 311, system ID 0765600 using Cobas c pack (reference number, 03001938322). C4 was analyzed through the Cobas c 311, system ID 0765619 using Cobas c pack (reference number, 03001962322).

### Heat stress

After the termination of the feeding experiment, five fish from each replicate were collected from the experimental glass aquaria and transferred into a 0.5-ton fiberglass tank containing 7 sub-rearing units (7-L perforated plastic cylindrical containers). Each container represents a replica of each treatment. Replicates A, B and C of each treatment have been exposed to the heat stress on the first, second and third days, respectively. Thus, the heat stress challenge was repeated three times over three consecutive days. The water temperature was gradually raised from 25 to 40 °C within 3 h. The water temperature was controlled by an artificial thermostat. After exposure to 40 °C for 4 h, fish from each unit were anesthetized using clove oil. The blood samples were collected to determine ALT, AST, IgM, C3 and C4, glucose and cortisol levels, as well as the hematological count of fish.

### Statistical analysis

Data of the growth performance and feed utilization were processed using one-way analysis of variance (ANOVA). Two-way ANOVA was used to evaluate the effect of heat stress and the experimental diets on fish. Duncan’s post hoc test was used to rank the means. *P* ≤ 0.05 was regarded as statistically significant. All statistics were processed using the SPSS package (version 23.0).

### Declaration

All methods are reported in accordance with the ARRIVE guidelines 2.0.

## Results

### Growth performance and feed utilization

Growth performance and feed utilization results of fish fed the experimental diets are presented in Table 2. The highest final weight (FW) and weight gain (WG) were obtained in fish fed CN100 followed by CN200 (*P* ≤ 0.05). Fish fed on CN50 gave moderate FW and WG and were similar to those fed on C100 (*P* ≥ 0.05). The lowest results of WG were recorded in fish fed CON, C50 or C200 (*P* ≤ 0.05). Fish fed on CN100 and CN200 showed the highest specific growth rate (SGR), followed by CN50 and C100 (*P* ≤ 0.05). However, SGR in fish fed CN200 did not significantly differ from CN50 or C100 (*P* ≥ 0.05). Fish groups of CON, C50 and C200 produced the lowest SGR (*P* ≤ 0.05). The highest result of average daily gain (ADG) was recorded in the CN100 group, followed by CN200 and then CN50 and C100 groups (*P* ≤ 0.05). Groups of CON, C50 and C200 showed the lowest values of ADG (*P* ≤ 0.05). The highest feed intake (FI) was recorded in CN50, CN100, CN200 and C100, followed by CON, C50 and C200 groups (*P* ≤ 0.05). The lowest FCR was detected in CN100 and CN200, followed by CN50 and C100, while the highest values of FCR were observed in CON, C50 and C200 (*P* ≤ 0.05). Groups of CN100 and CN200 gave the highest values of protein efficiency ratio (PER), followed by CN50, C100 while the lowest values were produced in CON, C50 and C200 (*P* ≤ 0.05).

**Table 2:** Effect of the experimental diets on the growth performance and feed utilization of Nile tilapia (*Oreochromis niloticus*).

Treatment	CON	CN50	CN100	CN200	C50	C100	C200
Initial weight (g)	13.21 ± 0.11	13.47 ± 0.24	13.87 ± 0.94	13.67 ± 0.24	13.90 ± 0.06	13.52 ± 0.28	13.17 ± 0.38
FW (g)	22.55 ± 0.30^e^	27.27 ± 0.17^c^	32.44 ± 0.99^a^	30.17 ± 0.44^b^	23.94 ± 0.54^d^	27.33 ± 0.33^c^	22.50 ± 0.29^e^
WG (g)	9.46 ± 0.34^d^	13.80 ± 0.14^c^	18.78 ± 1.20^a^	16.30 ± 1.35^b^	10.04 ± 0.60^d^	13.81 ± 0.60^c^	9.33 ± 0.40^d^
SGR (% day^-1^)	0.84 ± 0.03^c^	1.08 ± 0.02^b^	1.33 ± 0.07^a^	1.20 ± 0.13^ab^	0.83 ± 0.04^c^	1.08 ± 0.05^b^	0.82 ± 0.04^c^
ADG (g day^-1^)	0.145 ± 0.005^d^	0.212 ± 0.002^c^	0.289 ± 0.018^a^	0.250 ± 0.020^b^	0.154 ± 0.009^d^	0.212 ± 0.009^c^	0.144 ± 0.006^d^
FI	18.90 ± 0.78^b^	22.53 ± 0.50^a^	23.03 ± 0.45^a^	23.33 ± 0.35^a^	19.70 ± 0.92^b^	22.37 ± 1.01^a^	19.60 ± 0.40^b^
FCR	2.00 ± 0.15^a^	1.63 ± 0.02^b^	1.26 ± 0.18^c^	1.42 ± 0.10^bc^	1.97 ± 0.14^a^	1.62 ± 0.09^b^	2.11 ± 0.15^a^
PER	1.77 ± 0.16^ cd^	2.17 ± 0.01^bc^	2.86 ± 0.50^a^	2.52 ± 0.22^ab^	1.82 ± 0.14^ cd^	2.19 ± 0.13^bc^	1.69 ± 0.12^d^

Values in the same row with different superscripts are significantly different (*P* ≤ 0.05). Values ± standard deviation. All values are mean of three independent biological replicates (n = 3).

### Body chemical composition

Results of the proximate chemical composition of Nile tilapia fed the experimental diets are illustrated in Table 3. There was not significant variation in the dry matter among different groups (*P* ≥ 0.05). The highest values of crude protein (CP) were obtained in CN50, CN100, CN200 and C100, followed by CON, C50 and C200 (*P* ≤ 0.05); however, C50 was not significantly different from CN50, CN100 and C100 (*P* ≥ 0.05). All treatments had higher crude lipid ratio than CON (*P* ≤ 0.05). The highest results of ash were recorded in CON and C200, followed by C50 and the lowest results were observed in CN50, CN100, CN200 and C100 (*P* ≤ 0.05).

**Table 3:** Effect of the experimental diets on the whole-body proximate composition (on dry weight basis) of Nile tilapia (*Oreochromis niloticus*).

Treatment	CON	CN50	CN100	CN200	C50	C100	C200
Dry matter	32.41 ± 0.29	31.89 ± 1.16	32.77 ± 1.45	32.78 ± 1.20	33.23 ± 0.58	32.26 ± 1.12	32.06 ± 0.44
Crude protein	52.97 ± 0.02^c^	57.12 ± 1.57^ab^	56.55 ± 1.07^ab^	58.29 ± 0.16^a^	55.05 ± 2.20^bc^	56.44 ± 0.43^ab^	53.50 ± 1.31^c^
Crude lipid	22.54 ± 0.58^b^	26.53 ± 0.98^a^	26.09 ± 0.89^a^	26.48 ± 1.00^a^	26.94 ± 0.31^a^	26.48 ± 1.63^a^	25.66 ± 0.42^a^
Ash	18.91 ± 0.58^a^	14.83 ± 1.31^ cd^	15.59 ± 0.05^ cd^	14.19 ± 0.71^d^	16.09 ± 0.59^bc^	15.55 ± 1.09^ cd^	17.40 ± 1.07^ab^

Values in the same row with different superscripts are significantly different (*P≤ * 0.05). Values ± standard deviation. All values are mean of three independent biological replicates (n = 3).

### Hematological analysis

Table 4 shows the hematological analysis of fish after the feeding experiment at 25 °C and after heat stress at 40 °C. Results revealed that the experimental diets and heat stress significantly affected some hematological parameters (*P* ≤ 0.05). Experimental diets had no effect on the hematological counts (*P* ≥ 0.05) except for C200, which gave higher leukocytes and lower neutrophils counts compared to the other experimental diets (*P* ≤ 0.05). However, after heat stress, an increase in the number of platelets, MCV, MCH, leukocytes and neutrophils were detected, while the number of lymphocytes was reduced (*P* ≤ 0.05) and there was no effect on the other hematological parameters (*P* ≥ 0.05).

**Table 4:** Effect of the experimental diets and heat stress on the hematological counts of Nile tilapia (*Oreochromis niloticus*).

Treatment Indices	25 °C								40 °C							
	CON	CN50	CN100	C-N200	C50	C100	C200	SDM	CON	CN50	CN100	C-N200	C50	C100	C200	SDM
Leukocytes	34.99	32.77	36.51	32.41	38.02	38.22	50.56	6.45	49.86	53.01	57.44	53.39	54.11	61.05	80.39	11.17
Erythrocytes	2.21	2.00	1.92	1.86	2.04	2.05	1.98	0.15	1.86	2.03	1.56	1.82	1.91	1.89	1.86	0.25
HGB	5.90	5.70	5.25	5.70	5.40	5.65	5.95	0.50	5.40	6.55	4.50	6.30	5.45	6.55	5.75	0.83
HCT	29.10	31.75	32.25	31.20	30.50	29.95	28.25	2.70	31.80	33.45	26.50	31.65	28.95	32.55	30.15	3.79
MCV	131.60	158.30	167.55	169.05	150.15	146.00	142.70	17.24	171.75	165.50	168.00	172.30	153.35	172.50	162.20	11.97
MCH	26.65	28.35	27.40	30.65	26.60	27.60	30.00	2.27	29.00	32.70	28.95	34.55	28.50	34.75	30.95	3.38
MCHC	20.30	17.90	16.45	18.20	17.70	18.90	21.15	1.87	16.95	19.70	17.30	20.05	18.95	20.10	19.10	1.93
PLT	21.00	25.00	23.50	23.00	25.00	24.00	24.50	2.16	51.50	51.50	66.50	58.00	53.00	57.00	57.00	4.94
NEUT	5.70	5.30	5.85	5.30	5.90	6.75	15.05	3.53	17.60	16.90	17.55	16.55	17.15	20.40	36.15	7.11
LYMPH	83.25	84.10	83.75	82.10	85.15	81.90	74.30	3.64	71.45	71.50	71.60	73.80	71.45	66.76	49.35	8.32
MONO	1.80	1.10	2.45	2.15	1.25	0.80	0.35	0.99	1.00	0.40	1.25	0.55	1.15	1.05	0.75	0.37
EO	0.05	0.10	0.10	0.25	0.15	0.05	0.45	0.16	0.15	0.20	0.10	0.05	0.70	0.85	1.65	0.76
BASO	9.15	9.30	7.75	10.05	7.40	10.30	9.75	1.31	9.65	10.80	9.25	8.95	9.55	10.70	11.70	1.32
IG	0.05	0.10	0.10	0.15	0.15	0.15	0.10	0.086	0.15	0.20	0.25	0.10	0.00	0.25	0.40	0.17

Variation source	Heat stress	Diet	Interaction	Duncan HSD for diets												
				CON	CN50	CN100	CN200	C50	C100	C200						
Two-way ANOVA																
Leukocytes	⁎⁎⁎	⁎⁎	*	b	b	b	b	b	b	a						
Erythrocytes	ns	ns	ns	a	a	a	a	a	a	a						
HGB	ns	ns	ns	ab	a	b	a	ab	a	a						
HCT	ns	ns	ns	a	a	a	a	a	a	a						
MCV	⁎	ns	ns	a	a	a	a	a	a	a						
MCH	**	ns	ns	b	ab	b	a	b	ab	ab						
MCHC	ns	ns	ns	ab	ab	b	ab	ab	ab	a						
PLT	***	ns	ns	a	a	a	a	a	a	a						
NEUT	***	⁎⁎⁎	ns	b	b	b	b	b	b	a						
LYMPH	***	⁎⁎⁎	**	a	a	a	a	a	b	c						
MONO	ns	ns	ns	ab	ab	a	ab	ab	ab	b						
EO	ns	ns	ns	b	b	b	b	ab	ab	a						
BASO	*	ns	ns	ab	ab	b	ab	b	a	a						
IG	ns	ns	ns	a	a	a	a	a	a	a						

*SDM*, pooled standard deviation of the mean.

*P ≤ 0.05, **P ≤ 0.01, ***P ≤ 0.001, *ns* non-significant.

Significant differences are indicated by different letters (*P* ≤ 0.05).

### Biochemical blood analysis

Results of the biochemical blood analysis before and after the heat stress of Nile tilapia fed the experimental diets are shown in Table 5. There is a profound effect of both the diets and heat stress on the biochemical blood parameters of fish (*P* ≤ 0.05). ALT showed the highest activity in C200, followed by CON and C100 and then C50 and CN200, while the lowest values were obtained in CN50 and CN100 treatments (*P* ≤ 0.05). The highest activity of AST was given in C200, followed by C100 and CON treatments, followed by C50, while the lowest activity was detected in CN50, CN100 and CN200 treatments (*P* ≤ 0.05). However, AST value in CN200 was not significantly different from C50 (*P* ≥ 0.05). The highest IgM levels were obtained in C200, C100, CN100 and CN200 followed by CN50, while C50 and CON gave the lowest values (*P* ≤ 0.05). C200 showed the highest level of C3, followed by CN50 (*P* ≤ 0.05), while C100, CN100 and CN200 did not significantly differ from C200 or CN50 (*P* ≥ 0.05). C50 and CON gave the lowest values for C3 (*P* ≤ 0.05). The highest C4 values were found in C200 and CN200, followed by CN50 and then CN100 and C100, while C50 and CON gave the lowest results (*P* ≤ 0.05). C200 gave the highest value of cortisol, followed by CON, followed by C100 and then CN200 and C50 (*P* ≤ 0.05). The lowest results of cortisol were recorded for CN50 and CN100 (*P* ≤ 0.05). Glucose obtained in C200 and CON was higher than in the other treatments (*P* ≤ 0.05). An increase in ALT, AST, IgM, C3, C4, cortisol and glucose was observed after the heat stress (*P* ≤ 0.05).

**Table 5:** Effect of the experimental diets and heat stress on the serum biochemical indices of Nile tilapia (*Oreochromis niloticus*).

Treatment	25 °C								40 °C							
	CON	CN50	CN100	CN200	C50	C100	C200	SDM	CON	CN50	CN100	CN200	C50	C100	C200	SDM
ALT	67.33	35.33	37.67	59.67	47.67	58.67	146.00	5.92	158.00	104.33	103.33	122.33	139.00	180.33	290.33	10.59
AST	387.33	118.33	147.00	212.33	213.00	285.00	648.67	37.06	767.00	471.67	417.67	575.33	675.67	937.00	1856.0	86.33
IgM	0.82	1.30	1.88	1.87	1.11	1.84	1.72	0.43	1.88	2.34	2.92	2.89	1.95	2.87	2.79	0.46
C3	1.30	2.16	2.72	2.49	1.26	2.20	2.28	0.59	3.42	4.65	4.80	4.45	4.13	5.47	5.59	0.52
C4	2.47	3.56	3.66	4.02	3.23	3.61	3.80	0.59	5.23	6.83	6.20	7.13	4.83	5.70	7.71	1.05
Cortisol	11.23	3.47	4.43	7.57	8.17	9.08	12.28	0.95	47.03	12.20	11.57	18.55	17.59	27.42	67.12	3.15
Glucose	110.67	21.33	37.00	43.17	46.44	39.67	65.73	7.55	149.00	143.33	141.00	142.11	142.67	156.37	181.67	14.07

Variation source	Heat stress	Diet	Interaction	Duncan HSD for diets												
				CON	CN50	CN100	CN200	C50	C100	C200						
**Two-way ANOVA**																
ALT	⁎⁎⁎	⁎⁎⁎	⁎⁎⁎	b	d	d	c	c	b	a						
AST	⁎⁎⁎	⁎⁎⁎	⁎⁎⁎	b	d	d	cd	c	b	a						
IgM	⁎⁎⁎	⁎⁎⁎	ns	c	b	a	a	c	a	a						
C3	⁎⁎⁎	⁎⁎⁎	⁎	c	b	ab	ab	c	ab	a						
C4	⁎⁎⁎	⁎⁎⁎	⁎⁎	e	bc	cd	ab	e	d	a						
Cortisol	⁎⁎⁎	⁎⁎⁎	⁎⁎⁎	b	e	e	d	d	c	a						
Glucose	⁎⁎⁎	⁎⁎⁎	⁎⁎⁎	a	b	b	b	b	b	a						

*SDM*, pooled standard deviation of the mean.

*P ≤ 0.05, **P ≤ 0.01, ***P ≤ 0.001, *ns* non-significant.

Significant differences are indicated by different letters (*P* ≤ 0.05).

## Discussion

In the current study, feeding Nile tilapia on diets containing curcumin led to noticeably better growth results compared to the control diet. Furthermore, this study remarkably reveals the superiority of nano-curcumin over its free-form in improving the growth performance of Nile tilapia. In the current study, the best growth performance (WG, SGR and ADG) and feed utilization (FCR and PER) of Nile tilapia were achieved at 100 mg kg^−1^, followed by 200 mg kg^−1^ diet of nano-curcumin. This is in agreement with Mahmoud et al.^23^ who found that feeding Nile tilapia (*Oreochromis niloticus*) with 50 or 100 mg kg^−1^ diet of curcumin enhanced growth performance and feed utilization. They linked this improvement in the growth performance and feed utilization to improved activities of the digestive enzymes (amylase, protease, trypsin and lipase)^22,40^. In addition, curcumin can enhance other enzymes (Na+/K+-ATPase, intestinal alkaline phosphatase, gamma-glutamyl transpeptidase, and creatine kinase) that are responsible for nutrients degradation and assimilation^22^. Moreover, curcumin may work as a prebiotic, promoting gut flora, improving the intestinal digestion and absorption, resulting in improved overall health and growth in fish^40^. Positive effects of dietary supplementation of curcumin have been revealed in different fish species such as grass carp (*Ctenopharyngodon idells*)^41^, crucian carp (*Carassius auratus*)^22^, large yellow croaker (*Pseudosciaene crocea*)^42^, rainbow trout (*Oncorhynchus mykiss*)^17^ and Asian sea bass (*Lates calcarifer*)^43^.

As previously mentioned, there is superiority in the present study for curcumin nanoparticles over free-curcumin in enhancing the growth rate of Nile tilapia. In other words, a lower dose of nano-curcumin can enhance the growth performance of Nile tilapia and give the same effects as a higher dose of free-curcumin. For instance, 50 mg kg^−1^ diet of nano-curcumin gave same effects as 100 mg kg^−1^ diet of free-curcumin. In support, nano-curcumin showed a 60-fold increase in the biological half-life compared to free-curcumin in rat models^44^. Moreover, nano-curcumin shows higher systemic bioavailability in plasma and tissues compared to free-curcumin^30^. This may be due to the low solubility of free-curcumin in water; therefore, it forms aggregates and is susceptible to opsonization, while nano-curcumin dissolves completely in water without aggregations due to its zeta potential^27^.

The addition of free-curcumin, nano-curcumin, at the highest level (200 mg kg^−1^ diet) in the current study decreased growth performance and feed utilization compared to CON. In fact, curcumin is a polyphenol. It has been reported that low doses of polyphenols can bio-accumulate in body tissues where they are not completely absorbed in the intestine^45^. Polyphenols in high levels may cause growth regression^46^. This effect was lower in the nano-curcumin treatments, indicating that nano-curcumin may be safer than its free-form. In support, Lee et al.^47^ revealed the bio-safety of nano-curcumin utilization.

In the present study, curcumin and nano-curcumin increased the crude protein and lipid contents in the fish body. Similarly, the dietary supplementation with curcumin at levels of 50–200 mg kg^−1^ diet significantly improved the crude lipid and protein deposition in Nile tilapia muscles^23^. This could be related to the regulation of the intestinal microbiota, which enhanced the efficient nutrient utilization^23^. Another explanation would be the beneficial effects of curcumin on the activity of fish digestive enzymes including trypsin, lipase, and amylase^22^. Additionally, negatively charged particles, such as nano-curcumin, slow down the adsorption rate of serum proteins, resulting in longer circulation half-lives than positively charged particles^48^. This could explain why fish fed diets containing nano-curcumin had a higher crude protein content than fish fed free-curcumin diets.

Any increase in liver enzymes activity, such as ALT and AST, is a biological marker of liver damage^49,50^. In the present study, ALT and AST levels were decreased in the nano-curcumin groups either before or after the heat stress and to some extent in the free-curcumin groups, demonstrating the advantages of nano-curcumin (this was justified earlier in this discussion). In similar, curcumin can prevent hepatotoxicity in rats induced by hydrogen peroxide and reduce levels of ALT and AST enzymes^51^. Moreover, experimental results in mice with fibrosis caused by carbon tetrachloride (CCl4) showed that nano-curcumin significantly reduced ALT and AST levels^52^. Thus, the curcumin ability to reduce some blood markers (such as ALT and AST) indicates its ability as an anti-inflammatory agent during liver injury^53^.

Despite the protective effects of curcumin against liver damage in this study at a moderate concentration (100 mg kg^−1^ diet), adverse effects appeared at a higher concentration (200 mg kg^−1^ diet). Following the same pattern, curcumin also showed a concentration-dependent effect in the gian carp (*Cyprinus carpio*), which suffered from CCl-induced liver damage^54^. Similarly, curcumin was found to have dual effects on alcoholic liver injury in male rats depending on the concentration, as its protective effect was only obtained at a low concentration, but acceleration of liver injury was observed at a higher concentration^55^. Another study in rats indicated that a high concentration with long-term intake of curcumin can decrease body weight, accelerate oxidative stress and inflammation and stimulate liver injury while increasing AST and γ-GGT levels^56^.

This dual effect of curcumin may be linked to its ability to stimulate heme oxygenase-1 (HO-1) in non-toxic and toxic concentrations, and HO-1 induction has been found to be associated with the production of reactive oxygen species (ROS), suggesting a causal relationship^57^. Similarly, Cao et al.^58^ found that at lower concentrations, curcumin have beneficial effects by inducing antioxidant activities; however, higher concentrations increase cellular ROS levels, resulting in oxidative DNA damage in human hepatoma G2 cells.

Heat stress elevates stress indicators, such as cortisol and glucose levels. In the current study, the inclusion of curcumin (excluding the high dose of 200 mg kg^−1^ diet) or nano-curcumin in tilapia diets reduced both glucose and cortisol either before or after heat stress. The best cortisol levels were detected at CN50 and CN100. This is probably justified by the nature of curcumin as a polyphenol. Polyphenols have been shown to improve stress indicators cortisol and glucose^59,60^). These effects can also be attributed to the anti-oxidation properties of curcumin, which has been found to eliminate lipid radicals in the cell membrane and become a phenoxyl radical^14,61^. Moreover, curcumin lowers hepatic glucose level by increasing the glucose uptake through upregulating GLUT2, GLUT3 and GLUT4 gene expressions^62^. In support, Wei et al.^63^ revealed that dietary curcumin significantly reduced stress-induced cortisol by one-third in pigs.

However, in this study, a higher concentration of curcumin (200 mg kg^−1^ diet) led to a higher level of cortisol compared to the other treatments, including the control group. In parallel, C200 produced a higher level of glucose similar to that obtained by the control group. The same effect was reported in laying hens, where higher doses of curcumin did not reduce cortisol compared to lower concentrations that decreased cortisol levels before or after a high temperature exposure^64^. The authors linked this effect to the dual-action potential of curcumin, as it can act as an antioxidant and/or oxidant, depending on its dose^65^. The high concentration of peroxides increases the release of ROS that damage cells and tissues^65^.

In this study, the experimental diets had no effect on the hematological counts, except for C200 which gave higher leukocytes and lower neutrophils compared to the other experimental diets. An elevated count of leukocytes at a high level of curcumin (200 mg kg^−1^ diet) may emphasize the stressful status of fish at this concentration. Leukocytosis is directly proportional to the severity of the stress as well as the damage from stress that subsequently triggers the immune defense system^66,67^.

Temperature affects hematological parameters in fish^68^. In this regard, after the heat stress, platelets, MCV, MCH, leukocytes and neutrophils counts were raised while the number of lymphocytes decreased, and there was no effect on other blood parameters in the current study. In support, Grzelak et al.^69^ reported significant lymphopenia and neutrophilia in acutely stressed zebra fish (*Danio rerio*) compared to unstressed or control fish. Moreover, thermal stress results in hypoxia or anoxia^70,71^, and this effect was reported in the present study. In turn, it was found that hypoxia increases the number of leukocytes, platelets, and neutrophils and reduces the number of lymphocytes in red tilapia^72^. This may be linked to the elevated level of cortisol^72^. Neutrophilia and lymphopenia were apparent after treatment with cortisol in carp (*C. carpio* L.)^73^. Crucian carp (*Carassius carassius*) produced a high ratio of neutrophils to lymphocytes in the blood after being subjected to stress, which is closely related to elevated glucocorticoid levels^74^.

Immunoglobulin M and complements (C3 and C4) are major components of the innate immune system of fish, and they are among the first lines of immune system defence and play a crucial role in protecting fish health^75–78^. IgM and complements play an important role in protecting the animal body from infection during stress, as well as promoting the engulfment of apoptotic and injured cells^79–82^. In fact, before and after heat stress in the present study, levels of IgM, C3, and C4 increased with the dietary inclusion of curcumin. Moreover, nano-curcumin showed better improvement in these indicators than free-curcumin. The results presented herein for IgM, C3 and C4 highlight two facts: the low dose of curcumin is better, and the nano-formulation is more effective (which were justified earlier in this discussion). In support, curcumin induced the innate immune response of Nile tilapia at a dose of 50 mg kg^−1^ diet, but growth was inhibited at higher levels^23^. The present study found that the levels of IgM, C3, and C4 increased with elevated temperature, revealing the stimulation of the production of anti-inflammatory substances to reduce tissue damage. This is supported by the serum liver enzymes (ALT and AST) results in the present study. Heat stress is known to cause tissue damage and oxidative stress in fish; thus, it leads to apoptosis and cell death^83–85^. The short-term acute heat stress leads to up-regulation of C3 and C4^86^. Moreover, deficiency in the complement system has increased the vascular damage, vascular permeability and angioedema^87^. A gradual increase in plasma IgM levels was observed with increasing temperature (18, 23, 28, 33 °C) in Nile tilapia except for the highest temperature^10^. Similarly, rearing sea bass (*Dicentrarchus labrax*) at a high temperature (23 °C) increased their IgM levels compared to fish reared at 17 °C^88^. On the contrary, other studies revealed that HS decreased IgM, C3 and C4 levels in fish^89–91^. This discrepancy may be justified by the duration of heat stress, where IgM, C3 and C4 levels elevate under acute stress but decrease under chronic stress^9,10^. Blunt snout bream (*Megalobrama amblycephala* Yih) showed an increase in alternative complement activities and IgM levels after 6 h of heat stress exposure, which decreased after 12 h^9^.

In conclusion nano-curcumin has superiority over free-curcumin in improving the growth performance, stress indicators, non-specific immunity and heat stress resistance of Nile tilapia. Dietary supplementation of nano-curcumin at levels of 50 or 100 mg kg^−1^ diet was effective in improving the performance and mitigating the negative effects of heat stress, while doses of 100 or 200 mg kg^−1^ diet improved the growth performance of Nile tilapia. Thus, a dose of 100 mg kg^−1^ diet was effective for both growth performance and heat resistance ability.

## Data Availability

All data generated or analyzed during this study are included in this published article.
